# Modeling the Intention and Adoption of Wearable Fitness Devices: A Study Using SEM-PLS Analysis

**DOI:** 10.3389/fpubh.2022.918989

**Published:** 2022-07-06

**Authors:** Qing Yang, Abdullah Al Mamun, Naeem Hayat, Gao Jingzu, Mohammad Enamul Hoque, Anas A. Salameh

**Affiliations:** ^1^UKM Graduate School of Business, Universiti Kebangsaan Malaysia, Bangi, Malaysia; ^2^Global Entrepreneurship Research and Innovation Centre, Universiti Malaysia Kelantan, Kota Bharu, Malaysia; ^3^UCSI Graduate Business School, UCSI University, Kuala Lumpur, Malaysia; ^4^BRAC Business School, BRAC University, Dhaka, Bangladesh; ^5^College of Business Administration, Prince Sattam Bin Abdulaziz University, Al-Kharj, Saudi Arabia

**Keywords:** wearable fitness device, intention, adoption, conspicuous consumption, youth

## Abstract

Wearable fitness devices (WFDs) are prevalent personal technology that empowers the users' management and supervision of their personal health. The current study explored the impact of health consciousness, health motivation, perceived cost, compatibility, usefulness, and perceived technology accuracy with the intention to use the WFDs. Furthermore, the users' conspicuous consumption and intention promote the usage of WFDs. A cross-sectional and quantitative research design was utilized for the current study, followed by data collection through social media and a final analysis with 1,071 samples data. The data analysis was accomplished with the partial least square regression structural equation modeling. The findings of this study revealed that the users' level of health consciousness, perceived compatibility, usefulness, perceived cost, and technology accuracy significantly influenced the intention to use WFDs. However, the conspicuous consumption and intention indicated the support for the usage behavior of the WFDs. This behavior significantly moderated the relationship between the intention and usage behavior for the WFDs. This study contributed to the theoretical realm for prompting the intention to use the WFDs with personal protection motivation that depicts the coping strategy and technology level attributes that form the intention to use WFDs. The WFDs manufacturers should therefore focus on developing WFDs features that harness usage behavior among the adults. Developing the personal responsibility to reduce the burden of the healthcare system and taking care of personal health could promote the usage of the WFDs.

## Introduction

Currently, technology supports the users' continuous evaluation and monitoring of personal health. The global healthcare scenario requires individuals to take serious consideration of personal fitness and health monitoring to avoid chronic diseases and prevent COVID-19 contagion (1). Mobile technology with associated accessories allows users to monitor and gauge their fitness and medical conditions at personal discretion (2). Wearable fitness devices (WFDs) are the new smartphone equipments that facilitate the users in managing their health (3).

The market for wearable devices (WDs) is growing consistently and may have reached USD 30 billion by the end of 2022 (4). The market players invest in application development and penetrate the WD market with state-of-the-art smart fitness bracelets (2, 5). WFDs support the users in evaluating their personal health conditions based on the user's personal choice (6). The users could monitor a few of the features associated with using the WDs, such as daily walking footsteps, sleep quality, diet frequency, and diet nourishment quality (7).

The WFDs comprise different variations such as smartwatches, smart bands, and mobile phone-assisted smart gadgets that facilitate health monitoring (2). The typical products available in the Chinese market are FitBit, Apple Watch, Samsung Galaxy Watch, and Honor watch (2). The WFDs are used to estimate blood pressure, glucose monitoring, and heart rate, which enables users to understand health-related data in real-time (5). In addition, WFDs help users measure body temperature, heartbeat rate, blood pressure, and daily mobility, among others (2). These devices also allow the users to achieve personal health and a healthy lifestyle. Regular health monitoring assists users in controlling health risks and reducing the risks of emergencies, hospitalization, and life-threatening conditions (8).

Besides the latent benefits of the WFDs, the acceptance and use of the WFDs remain restricted, while the mass adoption is yet to be acknowledged (7). The WFD assists the management of personal healthcare and reduces the burden on the public healthcare system (9). The use of WFDs remains low compared to wearable medical devices (WMDs) (5). Although the average user of WMDs omits or does not use WFDs, there are still some users who use WFDs and WMDs on an ongoing basis (2). Manufacturers and marketers of the WFDs are led toward the understanding and creation of new attributes that allow them to achieve long-term usage and penetration of WFDs (10).

China has become the most populated nation worldwide. According to the seventh national census report released by the National Bureau of Statistics of China on May 21, 2021, the population of 0–14 years amounted to 25,3383,938 and accounted for 17.95%, while the population of 15–59 years amounted to 894,376,020 and accounted for 63.35%. Furthermore, the population of 60 years and higher amounted to 26,4018,766, which accounted for 18.70% (11). The growing population is putting pressure on the healthcare industry to grow and expand to meet the healthcare needs of an increasing number of people and to provide better healthcare facilities and treatments for the entire Chinese population (12). Receiving and monitoring personal healthcare with technology is an effective method to reduce the load on the public healthcare system and encourage individuals to manage their health at their convenience (13). The steady adoption of WFDs offers the acceptance of the technology by the Chinese users, which indicates the affluence, health consciousness (HCS), and motivation among the Chinese population (14).

The dynamics of the changing world require individuals to take care of their personal health with the assistance of technology. Furthermore, the current extended waves of the COVID-19 epidemic and the issue of prolonged lockdowns lead to the importance of the individual to take care of personal health and make every effort to reduce hospital visits. The available WFDs are suitable for the screening and management of personal health issues at the convenience of the home and reduce the possibility of the COVID-19 epidemic (13).

However, the use of WFDs reduces over time, indicating that the WFDs users do not make consistent use of WFDs due to personal reasons and the state of WFD features. Therefore, it would be noteworthy to investigate and demonstrate the formation of the intention and usage of WFDs with the health attitudes and technology level attributes. The current study explored the intention and adoption of WFDs with the factors of WFDs technology (e.g., HCS, health motivation (HMN), perceived computability, perceived cost (PCT), perceived technology accuracy (PTA), and perceived usefulness) within the growing adult population. This study used the Technology Acceptance Model (TAM) and the Unified Theory of Acceptance and Use of Technology (UTAUT) to explore the impact of health awareness, HMN, perceived computability, PCT, perceived technical accuracy, and perceived usefulness on the intention to use and adoption of WFD technology. In previous studies as well, it has been confirmed that HCS and HMN affect users' intention to use medical electronics (15, 16). In the past studies of the TAM model, perceived computability, PCT, perceived technical accuracy, and perceived usefulness as individual perceptual aspects influenced their perceptions of products and perceptions of using them (15, 17). UTAUT proved to be a better model not only because it contains more determinants, but also clarifies the factors that influence the purpose of users' actions and behavior. This study attempts to combine the TAM and UTAUT models to incorporate some factors that influence users' behavioral intentions and behaviors for research. The current study focuses on the Chinese region by examining the Chinese population to understand the intention and adoption of WFDs among the Chinese adult population.

## Literature Review

### Unified Theory of Acceptance and Use of Technology Model (UTAUT)

To understand the acceptance and use of a particular form of technology (such as WFDs), this study uses the UTAUT as a suitable and appropriate theoretical model to examine the acceptance and use of technology and the factors that influence user actions and behavioral purposes (15). Individual health behavior triggers protection motivation and health beliefs (6). Coping appraisal and threat appraisal are the significant aspects of protection motivation. Specifically, the coping appraisal is based on the notion of self-efficacy, response efficiency, and response cost to manage the health challenges (3). However, the threat appraisal relies on the perception of severity and vulnerability, and it is suitable to be employed as a theoretical domain to discuss the prevention behavior for health challenges or epidemic calamities (9). Furthermore, HMN and concern for personal health are valuable predictors for the adoption of coping strategies to avoid health issues and the use of technology for personal health (5). Technology attributes also encourage the adoption of health technologies to manage personal health (18).

### The Technology Acceptance Model

The TAM is one of the frameworks used in this study, and the use of this model can help to consider how different factors influence an individual's use and acceptance of a particular form of technology, such as a wearable activity tracker like the WFD (19). The TAM was used in this study to explore whether the perceived factors associated with a particular form of technology in a population affect their intention and behavior toward the use of a particular form of technology. To better understand the intention and adoption of WFDs, this study expands the TAM model by adding three components to the original TAM model: perceived computability, PCT, and perceived technical accuracy. Moreover, the technical attributes of usefulness, compatibility, cost, and accuracy are significant for developing the intention to employ health-related technologies (20). Conspicuous consumption depicts the personal desire to acquire the goods or services that contribute to the achievement through a bold display of the product (21). The use of technology by an adult is becoming a fashion that builds the individual's social standing and satisfies the innate status predispositions (22).

### Hypotheses Development

#### Health Consciousness (HCS)

HCS denotes the realization that health is vital for a thriving and prosperous life. Health awareness builds the innate necessity that health is vital, and taking care of personal health could support an individual to show a good performance in aspects of life (23). The HCS refers to the personal belief that taking care of personal health is imperative, and individuals make a personal effort to maintain their personal health conditions (5). HCS develops the sense of urgency and necessity to take care of personal health and take the necessary efforts to maintain personal health conditions (9). Technology-based health services with a higher degree of personal HCS are mainly adopted. The technology empowers the individuals to offer the necessary support for daily health examination and management at the user's convenience (8). Sergueeva et al. (10) documented a significant influence on HCS using healthcare technology. Based on the existing evidence, the following hypothesis was developed:

H_1_: HCS has a positive effect on the intention to use WFD.

#### Health Motivation (HMN)

Motivation triggers the need to engage in a specific behavior. Individuals who value personal health should perform the necessary actions to avoid personal health challenges (24). HMN is described as the personal predisposition to value personal health and attempt to learn new knowledge to maintain health conditions (14). This motivation encourages individuals' daily routines and builds the necessity to engage in health practices. Personal HMN builds the need to learn new knowledge and practices to prevent injuries and confidently engage in health maintaining attitude (24). HMN has a substantial impact on behavioral intentions, which in turn affects the actual behavior (25). Consumer motivation has been shown to influence purchase intentions and behavior in previous studies, implying that consumers' personal motivation could influence their intentions and consequently actual behaviors (25–27). Accordingly, the following hypothesis was proposed:

H_2_: HMN has a positive effect on the intention to use WFD.

#### Perceived Compatibility (PCM)

In previous studies, compatibility has been considered as a perceived characteristic of predicting innovation technology acceptance behavior and has demonstrated the importance of compatibility in predicting individual technology acceptance, implying that compatibility can influence individual technology acceptance and intention to use the technology (28, 29). Good technology should be compatible with the users' daily routines and needs (30). The perspective users require the healthcare technology to be compatible with their lifestyle, daily work, and life routine. Given that the management of health is a daily routine, users aim for the healthcare technology to be in sync with their lifestyle and work agenda (31). The healthcare technology compatibility creates mental satisfaction and nurtures the perspective of users in using the wearable healthcare technology (1). Following the empirical evidence, the following hypothesis was proposed:

H_3_: PCM has a positive effect on the intention to use WFD.

#### Perceived Cost (PCT)

The PCT was specified as the monetary and non-monetary cost attached to the technology's possible use (17). The technology prices at the market entry point are significantly higher than the existing technologies, while the novel technology prices reduce the inclination of employing the technology (32). The use of technology constantly requires specific knowledge and skills for using technology (33). Technology prices and lack of specific knowledge increase the PCT for the prospective users (34). Notably, the healthcare technologies are high priced at the initial stage of market entry, thus restricting the users' intention to use the healthcare technology (9). Users' behavioral intents and behaviors can be influenced by PCTs, according to past studies (35, 36). In conclusion, it is possible that there is a link between PCT and consumers' intention to use and behavior, which leads to the following hypothesis:

H_4_: PCO has an effect on the intention to use WFD.

#### Perceived Usefulness (PUF)

The perception of technology's usefulness results in ease and encourages users to perform daily activities efficiently and at a fast rate (34). The perception of usefulness is based on the benefits for the user in saving time and effort (37). The usefulness of technology is the vital factor that promotes the intention to use the technology, while the usability of technology harnesses the same intention (3). In the case of healthcare technologies, ease of use and healthcare technology save the users' time and effort, and positively influence the intention to use the healthcare technology (38). Wang et al. (1) highlighted the usefulness of healthcare technology in encouraging the intention to use this technology. Accordingly, the following hypothesis was proposed:

H_5_: PUF has a positive effect on the intention to use WFD.

#### Perceived Technology Accuracy (PTA)

Technology precision reflects the technology's ability to consistently show a good performance and accurate results for the users (39). PTA denotes the degree of users' confidence that the technology could accomplish the promised tasks (13). Notably, PTA is a significant interpreter of customers' satisfaction and intention to use the technology (5). The PTA is built with the reliability and confidence that technology could offer standard results and promote a positive attitude toward technology use (1). Alam et al. (5) have demonstrated that PTA can directly influence the intention of users to use and adopt e-health-based products. In many studies, it has been confirmed that PTA has a significant impact on the intention to use and actual adoption behavior of e-health products (15, 40). Therefore, the following hypothesis was developed:

H_6_: PTA has a positive effect on the intention to use WFD.

#### Intention to Use a Wearable Fitness Device

The intention to use a particular technology as a core factor in TAM and UTAUT models has been the subject of many scholarly studies. Behavioral intention can also be understood as a self-reported degree of propensity to use a new technology in the future (1). Intention to use a technology can also be used to predict the actual use of a specific technology by users (5, 15). In most Theory of Reasoned Action (TRA) and Theory of Planned Behavior (TPB)-related research frameworks, there is a clear association between users' intention to use and actual behavior and intention to use predicts users' actual behavior, which in turn influences actual actions (15, 41, 42). Adults with health conditions require healthcare technologies to monitor and manage their personal health. The intention to use the healthcare device promotes the use of healthcare technology devices (33). Besides, stronger intention nurtures the use behavior toward healthcare technology. The following hypothesis was developed:

H_7_: Intention to use WFD has a positive effect on the adoption of WFD.

### Mediating Effect of Intention to Use WFD

The behavioral intention remains the significant predictor of actual adoption behavior. Higher HCS and motivation lead to the inclination to use healthcare technologies in the form of e-health services, fitness devices, and medical devices to maintain personal health (5). HCS and motivation are the particular preferences that incentivise health behavior through intention (8, 24).

The technology level attributes of compatibility, cost, usefulness, and accuracy remain the meaningful predictor of the intention to use and usage of the technology (3, 37). In the case of personal healthcare technologies, the features of users' compatibility, cost and usefulness, and the perception of technology accuracy nurture the healthcare technology adoption (1). Accordingly, the following mediating hypotheses are developed:

H_8_: Intention to use WFD positively mediates the effect of HCS, HMN, PCM, PCT, PUF, and PTA on the usage of WFD.

### Moderating Effect of Conspicuous Consumption

Technology users are inclined to openly demonstrate that users are technology savvy and able to use high-priced technology products (22). A conspicuous consumer attitude elaborates that the prospective consumer is involved in the use of technology to gain respect and embody the honor with technology (21). The use of novel technology contributes to individuals' popularity and helps deliver a personal statement of the individuals to others (3). Many young consumers are inclined to use the new technology to be acknowledged by others and gain popularity among their peers (21, 30). Currently, most of the research on conspicuous consumption has focused on the purchase behavior of high-end branded products and luxury goods for consumers, which in turn provides insights into consumer intent, consumption behavior, and consumption preferences (43–46). The relationship between conspicuous consumption and consumers' personal intentions has been studied in previous research in different cultural settings, where consumers and users were able to gain social status and subjective well-being through conspicuous consumption, which in turn generated pride and a need for uniqueness (43). The personal conspicuous consumption inclination creates the necessary intention of being engaged in technology use.

Previous studies have examined the relationship between consumers' or users' intention to use and use behavior by using conspicuous consumption as a mediator. Most studies have confirmed that conspicuous consumption can influence consumers' intention to consume or consumption behavior by examining consumers' personal subjective factors such as personal perception, value perception, and personal needs (43–46). In studies on consumer perceived performance and satisfaction, conspicuous consumption as a mediator has been shown to moderate the relationship between consumers' perceptions of products and consumer satisfaction (1, 47). Since the relationship between conspicuous consumption and consumer intention to use has been established in previous studies, conspicuous consumption as a mediator is examined in this study to understand whether conspicuous consumption as a mediator can influence the adoption behavior of consumers (or users) of the WFDs. In this context, the following hypothesis was proposed:

H_9_: The relationship between the intention to use the WFD and usage of WFD is positively moderated by conspicuous consumption.

**Figure 1:**
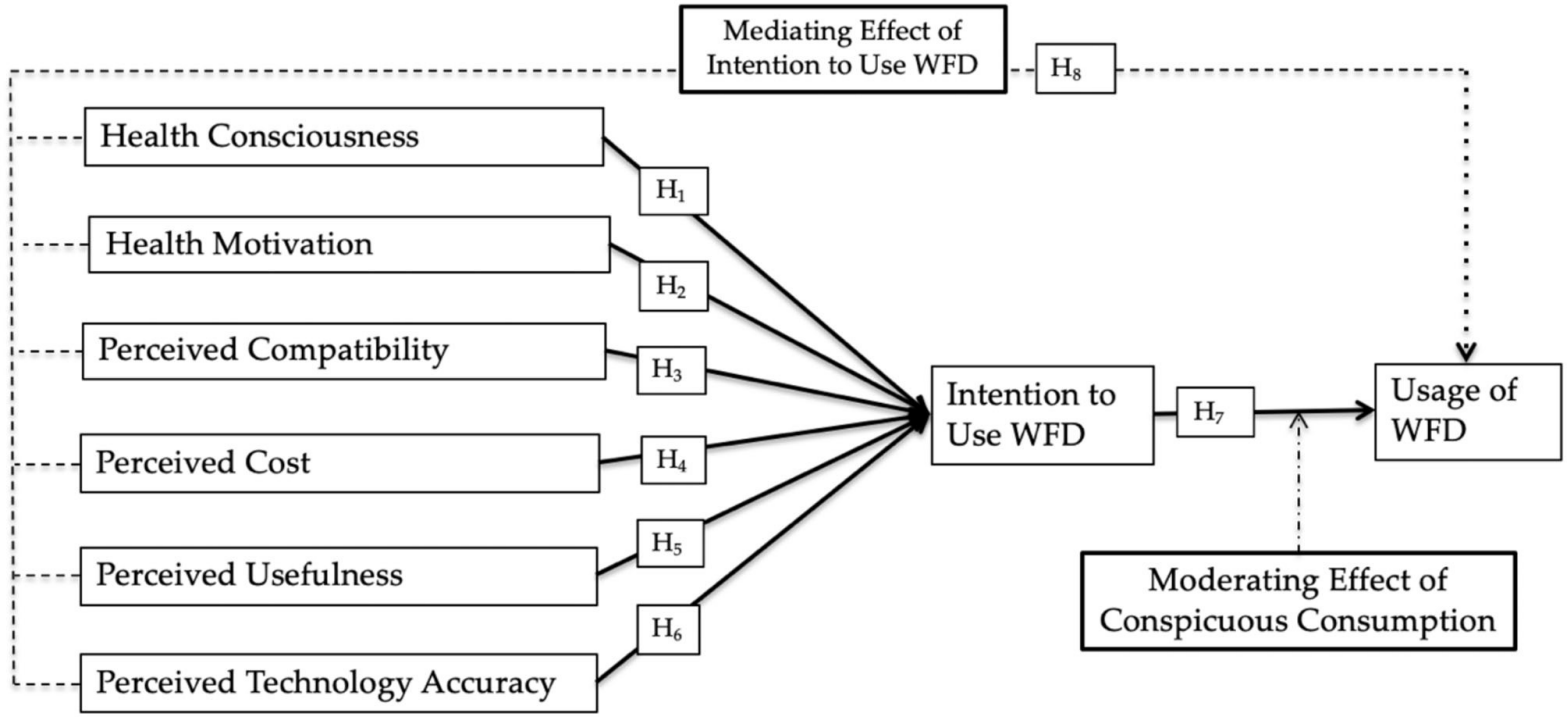
Research framework.

The proposed theoretical framework of this research is presented in Figure 1.

## Research Methodology

### Sample Size Calculations and Data Collection

The population in this study comprised Chinese adults. The sample size estimation was performed with the G-Power 3.1 for the power 0.95 and effect size 0.15 with eight input variables. The analysis facilitated the collection of a minimum of 160 effective samples to achieve the targeted level of analysis with the required power and effect size (48). This study required a minimum of 200 sample sizes to conduct the PLS-SEM analysis (49). The online questionnaire used in this study was distributed *via* WJX. The online questionnaire was then distributed through Chinese social media such as WeChat, forums, QQ, and Weibo using convenience sampling techniques. Data collection took place between February 27, 2022 and March 6, 2022, with a final screened sample of 1,074 valid samples retained.

### Measurement Scales

The scale for the study was developed from well-known and esteemed scales. The question items were utilized to measure the variables and relevant sources presented in Supplementary Appendix 1. The final scale was developed in English and subsequently translated into Chinese. Two Chinese language experts evaluated the final version of the developed scale to estimate the accuracy and effectiveness of the questionnaire items and obtain appropriate responses from the prospective study respondents (50). All the questionnaire items relating to all variables except the usage of WFDs were measured using a seven-point Likert scale (1: strongly disagree, 7: strongly agree), whereas endogenous variables (actual usage of WFDs) were graded based on a seven-point Likert scale (how often do you use WFDs? 1: never, 7: everyday).

### Common Method Variance (CMV)

The first Harman's one-factor test was adopted to investigate the CMV issue as an indicative procedure (51). The single factor accounted for 43.85%, indicating no severe issue of CMV as the single factor extraction was <50%. The second test employed for the current study was the full collinearity test for all the study constructs. This test was suggested by Kock (52). Overall, all the study variables were regressed on the new variable created from the study constructs. The variance inflation factor (VIF) for the regression analysis was evaluated. Moreover, the VIF scores for all the input variables must be <3.3 to establish the non-existence of the multi-collinearity issue in the data. The full collinearity test results suggested that no issue was present in the multi-collinearity in the data, given that the VIF values for HCS, HMN, PCM, PCT, perceived usefulness, PTA, conspicuous consumption, intention to use WFDs, and usage of WFDs amounted to 2.136, 2.639, 2.723, 2.033, 2.955, 2.809, 2.438, 2.773, 2.769, and 2.769, respectively.

### Data Analysis Method

This study applied the PLS-SEM technique to verify the proposed model and examine the proposed hypotheses using SmartPLS 3.0. Notably, many studies have validated and used the PLS-SEM technique for hypothesis testing (49). The characteristic of this technique is its flexibility regarding data allocation, which is suitable for a small size sampling (52). Besides, the inspection of the construct reliability, convergent validity, and discriminant validity before examining the structural model is important (53). Furthermore, Cronbach's Alpha measured the items' reliability, while Dijkstra-Hensele's *rho*, composite reliability, and the average variance extracted (AVE) measured the internal consistency reliability (49). Discriminant validity was examined by the Fornell–Larcker criterion, heterotrait–monotrait ratio (HTMT), and loadings and cross-loading. The path coefficients were used to test the hypothesis (53) with the use of beta (coefficients), confident interval, *t*-value, and *p*-value (49).

## Findings

### Demographic Profile of Respondents

As presented in Table 1, the respondents' demographic demonstrated that 51.3% of the respondents were male, while the rest of the respondents were female. Most of the respondents (30.7%) were aged between 23 and 26 years old, 23.1% were aged between 27 and 30 years old, 20.9% of the respondents were aged between 18 and 22 years old, and 19.6% of the respondents aged above 31 years old. Following this, 8% of the respondents originated from Beijing, 11.6% lived in Shanghai, 5.6% of the respondents lived in Guangdong, 6.6% lived in Guangxi, 8.4% lived in Zhejiang, 8.5% lived in Shandong, 5.4% lived in Hunan, 10.1% lived in Jiangsu, and the rest of the respondents lived in other provinces.

**Table 1:** Demographic characteristics.

	**N**	**%**		**N**	**%**
**Gender**			**Education**		
Male	551	51.3	Secondary school certificate	120	11.2
Female	523	48.7	Diploma	242	22.5
Total	1074	100	Bachelor degree or equivalent	476	44.3
			Master's degree	180	16.8
**Age**			Doctoral degree	56	5.2
18 or Less than 18 years	61	5.70	Total	1074	100
18-22 years	225	20.9			
23-26 years	330	30.7	**Average monthly income**		
27-30 years	248	23.1	Below CNY 2500	225	20.9
31 years or above	210	19.6	CNY 2501- CNY 5000	236	22.0
Total	1074	100	CNY 5001- CNY 7500	233	21.7
			CNY 7501- CNY 10,000	162	15.1
**Living province**			CNY 10,001- CNY 12,500	98	9.1
Beijing	86	8.0	More than CNY 12,500	120	11.2
Shanghai	125	11.6	Total	1074	100
Guangdong	60	5.6			
Guangxi	71	6.6			
Zhejiang	90	8.4			
Shandong	91	8.5			
Hunan	58	5.4			
Jiangsu	108	10.1			
Others	385	35.8			
Total	1074	100			

The majority of the respondents (44.3%) had completed Bachelor's level education, 22.5% had completed diploma-level education, 16.8% had fulfilled Master's degree, and 11.2% had obtained secondary school certificates. Most of the respondents (22.0%) had an average monthly income between CNY 2,501 and 5,000, 21.7% of the respondents had an average monthly income of CNY 5,001 and 7,500, 20.9% of the respondents had an average monthly income of less than CNY 2,500, 15.1% of the respondents had the average monthly income between CNY 7,501 and 10,000, and 11.2% of the respondents had the monthly income of higher than CNY 12,500.

### Reliability and Validity

In the first stage of the PLS-SEM analysis, Cronbach's alpha (CA), composite reliability, and rho-A were utilized to evaluate the internal reliabilities. The internal reliabilities scores for these three measures proved that all the constructs achieve satisfactory internal reliabilities. The minimum score for CA amounted to 0.741, CR was 0.837, and Dijkstra-Hensele's *rho* amounted to 0.744. The convergent validity was assessed with the AVE measure. Notably, the AVE score for every construct must exceed 0.50 to confirm the convergent validity. The outcome demonstrated that all AVE scores had sufficient convergent validity (49).

The issue of multicollinearity was evaluated with the VIF for each construct. As a result, all the VIFs were found to be lower than 3.3 (53). Given that the values of VIF of all variables were lower than 3.3, no issue of multicollinearity was found (52). Furthermore, the findings of the CA, CR, rho_A, AVE, and VIF are presented in Table 2. Following that, discriminant validities were determined with the Fornell–Larcker criteria, HTMT ratio, and cross-loading table. Fornell–Larcker criterion is based on the square root of AVE for each construct and the correlation with other constructs. The square root of AVE for a construct should be higher than the correlations. The findings suggested that every construct has sufficient discriminant validity. Moreover, the HTMT ratio score should be lower than 0.900 to establish the discriminant validity for each construct (53). Overall, the result indicated that all the study constructs could achieve sufficient discriminant validity. The loading and cross-loading table confirmed that discriminant invalidities were not present in the study constructs. The results are presented in Supplementary Appendix 2.

**Table 2:** Reliability and validity.

**Variables**	**No. of items**	**Mean**	**Standard deviation**	**Cronbach's alpha**	**Dijkstra-Hensele's *rho***	**Composite reliability**	**Average variance extracted**	**Variance inflation factors**
HCS	4	5.462	1.122	0.804	0.809	0.872	0.631	2.173
HMN	4	5.331	1.131	0.815	0.816	0.878	0.643	2.728
PCM	5	5.160	1.130	0.863	0.864	0.901	0.647	2.785
PCT	5	4.911	0.990	0.758	0.758	0.837	0.507	2.069
PUF	3	5.158	1.132	0.778	0.780	0.871	0.693	2.663
PTA	3	5.110	1.094	0.741	0.744	0.853	0.659	2.447
CCM	4	5.048	1.105	0.822	0.824	0.882	0.652	1.959
IWFD	4	5.162	1.067	0.842	0.842	0.894	0.679	1.967
UWFD	1	5.020	1.412	1.000	1.000	1.000	1.000	–

*HCS, Health consciousness; HMN, health motivation; PCM, perceived compatibility; PCT, perceived cost; PUF, perceived usefulness; PTA, perceived technology accuracy; CCM, conspicuous consumption; IWFD, intention to use WFD; UWFD, usage of WFD*.

*Author's data analysis*.

### Hypothesis Testing

The adjusted *r*^2^ value for the six exogenous constructs (e.g., HCS, HMN, PCM, PCT, perceived usefulness, and PTA) on the intention to use the WFD explicates 61.8% of the variance in the intention to use the WFD. The predictive relevance (*Q*^2^) score for the part of the model amounted to 0.416, which represented a large predictive relevance (49). The adjusted *r*^2^ value for the conspicuous consumption and intention to use the WFD as input constructs on the use of WFDs represented 44.2% of the variance in the use of WFDs. Meanwhile, the model fragment predictive relevance (*Q*^2^) score was 0.436, which indicated a considerable predictive relevance (49).

**Figure 2:**
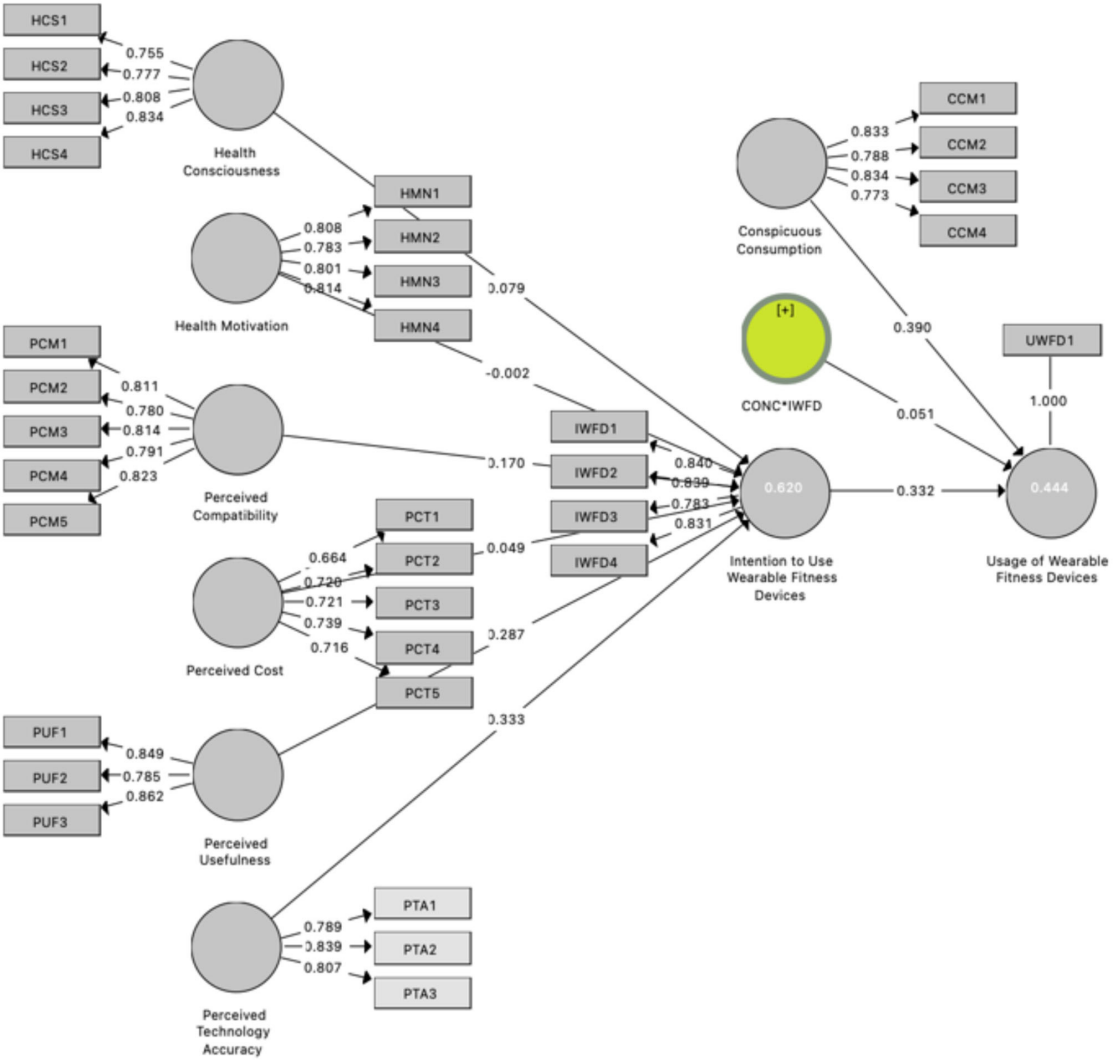
Structural model with path weights.

The path coefficient of HCS on the intention to adopt fitness devices achieves the acceptable *p*-value, which presents the evidence to accept the H_1_. The path value of HMN on the intention to adopt the fitness device is not statistically significant, therefore rejected H_2_. Following that, the path value between the PCM on the IWD could lead to the significance level and the advocate acceptance of H_3_. Consecutively, the path value between PCT and IWD is positive, and offers statistical provision to accept the H_4_. The path score for PUF on the IWD is positive and statistically significant, therefore accept H_5_; which is followed by the acknowledgment of the path coefficient between PTA and IWD regarding the statistical provision to accept the H_6_. Finally, the path coefficient between IWD and the UWFD supports the argument that the intention to use the WFD significantly influences the use of the WFD and accepts the H_7_. The results of path analysis are presented in Table 3 and Figure 2.

**Table 3:** Path coefficients.

**No**.	**Path**	**Beta**	**CI – min**	**CI – max**	***t***	***p***	***r^2^***	***Q^**2**^***	***f^2^***	**Decision**
***Factors affecting intention to use WFD***										
H_1_	HCS → IWD	0.079	0.025	0.133	2.377	0.009			0.007	Supported
H_2_	HMN → IWD	−0.002	−0.066	0.056	0.061	0.476			0.000	Rejected
H_3_	PCM → IWD	0.170	0.112	0.230	4.648	0.000	0.620	0.416	0.027	Supported
H_4_	PCT → IWD	0.049	0.006	0.097	1.755	0.040			0.003	Supported
H_5_	PUF → IWD	0.287	0.210	0.353	6.534	0.000			0.082	Supported
H_6_	PTA → IWD	0.333	0.264	0.411	7.300	0.000			0.119	Supported
**Factors affecting usage of WFD**										
H_7_	IWD → UWFD	0.332	0.266	0.403	8.093	0.000	0.444	0.436	0.101	Supported

*HCS, Health consciousness; HMN, health motivation; PCM, perceived compatibility; PCT, perceived cost; PUF, perceived usefulness; PTA, perceived technology accuracy; CCM, conspicuous consumption; IWFD, intention to use WFD; UWFD, usage of WFD*.

*Author's data analysis*.

### Mediation Analysis

The mediation analysis results (as presented in Table 4) confirm that the intention to use WFD insignificantly mediates the relationship between HMN and usage of WFD. However, the path from HCS, PCM, PCT, perceived usefulness, and PTA on the usage of WFD is significantly mediated by the intention to use the WFD.

**Table 4:** Mediating effect.

**No**.	**Path**	**Beta**	**CI – min**	**CI – max**	***t***	***p***	**Decision**
***Mediating effect of intention to use WFD***							
HM_1_	HCS → IWD → UWFD	0.026	0.008	0.047	2.196	0.014	Accept
HM_2_	HMN → IWD → UWFD	−0.001	−0.022	0.018	0.060	0.476	Reject
HM_3_	PCM → IWD → UWFD	0.056	0.036	0.080	4.128	0.000	Accept
HM_4_	PCT → IWD → UWFD	0.016	0.002	0.032	1.708	0.044	Accept
HM_5_	PUF → IWD → UWFD	0.095	0.064	0.126	5.279	0.000	Accept
HM_6_	PTA → IWD → UWFD	0.110	0.077	0.150	5.001	0.000	Accept

*HCS, Health consciousness; HMN, health motivation; PCM, perceived compatibility; PCT, perceived cost; PUF, perceived usefulness; PTA, perceived technology accuracy; CCM, conspicuous consumption; IWFD, intention to use WFD; UWFD, usage of WFD*.

*Author's data analysis*.

### Moderation Analysis

**Figure 3:**
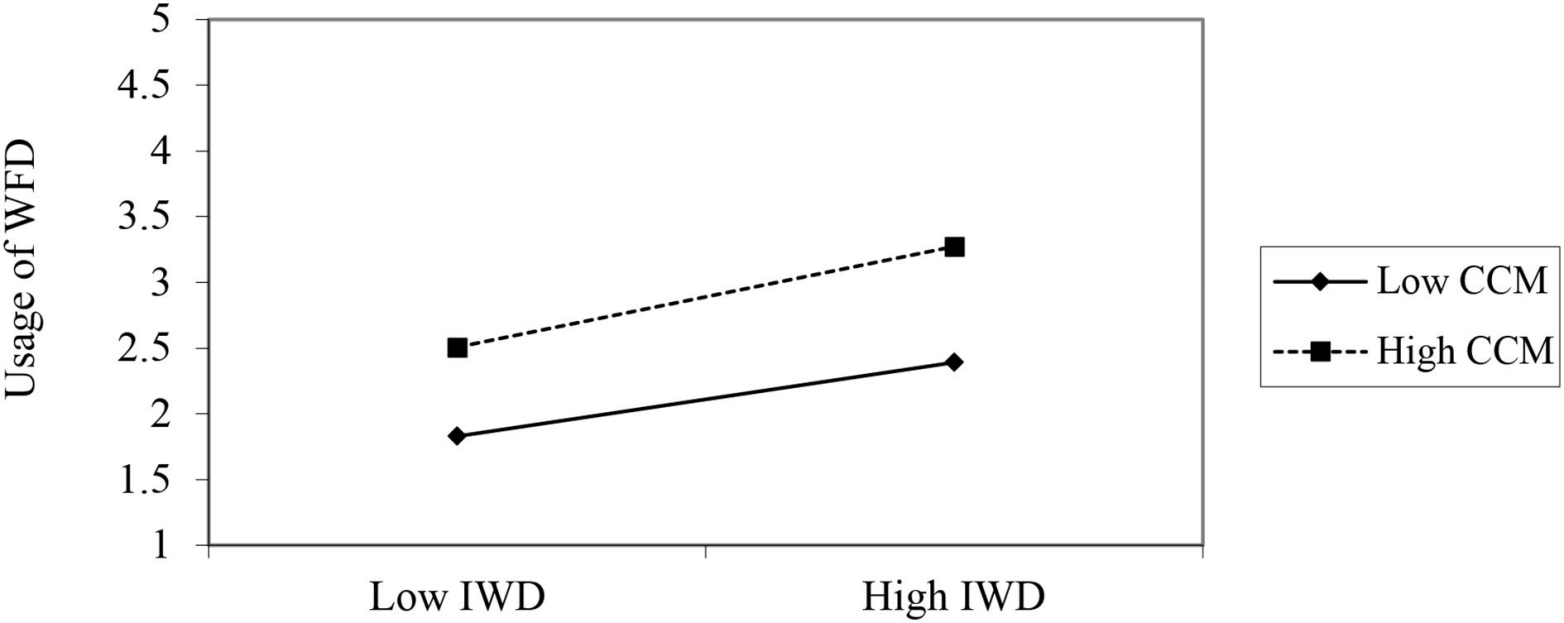
Moderating effect.

In the current study, it was suggested that conspicuous consumption interacted with the intention to use a WFD and significantly moderated the usage behavior of WFDs. The analysis suggested that the CCM significantly moderated the relationship between the IWD and the UWFDs. The moderation analysis findings are presented in Table 5 and Figure 3.

**Table 5:** Moderating effects.

**Path**	**Coefficients**	**CI – min**	**CI – max**	***t***	***P***	**Decision**
CCMxIWD → AWD	0.051	0.014	0.092	2.140	0.016	Full moderation

*CCM, Conspicuous consumption; IWFD, intention to use WFD; UWFD, usage of WFD*.

*Author's data analysis*.

## Discussion and Conclusion

The current work explored and demonstrated the intention and adoption of WFDs among Chinese adults. Following the cross-sectional data used for the analysis, the results suggested that HCS influenced the intention to use WFDs. Overall, the current result was in line with the results presented by Sergueeva et al. (10), in which consumers' HCS substantially influenced their intention to use the WFDs.

The study result suggested that the respondents' HMN had an insignificant impact on the Chinese intention to use WFDs. It was also found that the Chinese consumer did not have the HMN to use WFDs. Overall, the findings illustrated that young adults did not possess the right HMN to engage in the WFDs. The lack of HMN as a personal trait may reduce the inclination to use the WFDs (14).

The study results suggested that PCM significantly influenced the intention to use WFDs. It was also suggested that Chinese consumers considered technology compatibility a necessary condition related to the intention to use WFDs. Overall, the results were in line with the results by Wang et al. (1) that mobile technology's PCM supported the consumers' intention to use the mobile technology for personal healthcare.

The study results demonstrated that the perception of associated costs significantly impacted the intention to use WFDs. It was indicated that Chinese consumers were willing to use WFDs due to their perception that the cost of the WFDs was relatively lower. This condition led to the inclination to use WDs. Overall, the results were in line with the results by Beh et al. (9), in which consumers used WDs when the value of the devices was found to be higher than the associated prices.

The perception of usability strongly supports the consumers' favorable attitude toward the adoption of technology. The current study confirmed that the perceived usefulness significantly affected the intention to use WFDs. Furthermore, it was indicated through the perception of the prospective consumers that technology usability is a significant predictor of the intention to use technology. Subsequently, the verdict matched the results assumed by Wang et al. (1) that the wearable healthcare device usability was crucial for the intention to use the wearable healthcare device.

The study result suggested that the PTA strongly impacted the Chinese consumers' intention to use WFDs. It was also emphasized that Chinese consumers considered technical accuracy a critical factor in the intention to use WFDs. Overall, the results were in line with the results presented by Alam et al. (5), in which the mobile health services were adopted by the consumer upon the acknowledgment that mobile healthcare technology showed result accuracy. The PTA built the consumers' confidence and offered consistent results for healthcare users.

The study results highlighted that the consumers' conspicuous consumption attitude suggested the implementation of user behavior for a WFD. Therefore, it was proven that conspicuous consumption is an essential predictor of user behavior for the study samples. Overall, the results were in line with the result presented by Goenka and Thomas (22), in which conspicuous consumption attitude promoted use behavior for innovative technology.

The study analysis confirmed that the intention to use the WFDs suggested the use behavior toward WFDs. This result was in line with Aksoy et al. (20), in which the consumers with stronger intention toward wearable healthcare devices nurtured use behavior.

The mediation result confirmed that the path between HCS and the use of WFDs was mediated by the intention to use the WFDs. The intention to use WFDs did not suggest the mediation of the relationship between HMN and the use of WFDs. The mediational analysis also indicated that the intention to use WFDs meaningfully mediated the relationship between technology level factors (e.g., PCM, PCT, usefulness, and technology accuracy) and the usage of the WFDs.

Finally, the moderation analysis result suggested that the relationship between the intention to use the WFD and user behavior for the WFDs was significantly moderated by conspicuous consumption. Young adults are interested in using novel technologies that empower individuals to gain respect and popularity among their peers. The perspective users with a conspicuous consumption attitude develop an inclination to start using the WFD.

### Theoretical Implications

The current work contributed to the theoretical realm for prompting the intention to use the WFDs with personal protection motivation that depicts the coping strategy and technology level attributes that form the intention to use WFDs. The intention and conspicuous consumption showed support for the user's behavior of the WFDs. Furthermore, the study demonstrated that reliability and price values were significant predictors of behavioral intention for young consumers in China. However, the moderating effect of personal income and pre-existing conditions did not influence the adoption of WFD. The integrated research illustrated that the model possessed the preeminent exploratory power to explore intention formation and adoption of WFDs among young adults.

It was found that the adoption of WFDs was mainly based on adults' conspicuous consumption attitude, which indicated the inclination of Chinese adults to use the WFDs for fashion or stature reasons besides health reasons. Young adults possessed lower HMN and consciousness to use the WFD and to gauge and manage their personal health in the period of COVID-19. Health management is a vital issue, and young consumers are looking for innovative technologies to harness healthcare behavior (5, 21).

### Practical Implications

The current study findings offered imperative practical inferences for the healthcare industry. Conspicuous consumption is a more significant interpreter for the usage behavior compared to intention and other technical factors. Healthcare industry management needs to understand that Chinese consumers are more inclined to use WFDs for visibility and prestige reasons. The marketer should highlight the usability of WFDs and concentrate on reducing the prices of WFDs (12). The WFDs usability could be enhanced by developing visual guidance that facilitated the learning and understanding of the WFDs features, usage, and the methods of obtaining the maximum benefits from the use of WFDs (6).

The development of the personal inclination among young individuals of taking care of their personal health using relevant wearable technology empowers WFDs users. This feature helps reduce the burden on the public healthcare system (2). Furthermore, promoting personal HCS and HMN encourages the right attitude toward the use of WFDs. A noteworthy finding of the study was that the conspicuous consumption attitude significantly contributed to the usage behavior of the WFDs. It also indicated that WFDs users are inclined to use the WFDs for visibility and prestige reasons rather than personal health concerns (10). The WFDs manufacturers are required to develop the WFDs features that harness usage behavior among the adults (4). Essentially, developing the personal responsibility to reduce the burden of the healthcare system and taking care of personal health could develop the usage behavior of the WFDs (12).

### Limitations

With the current research creating academic and applied implications for scholars, the healthcare industry, and users, the current study was associated with four limitations. First, the current study primarily utilized the quantitative research design to offer a limited understanding of the phenomenon under study. Therefore, future research is required to utilize the mix-method research design to gain a complete insight into the intention and adoption of WFDs. Second, the current work employed the limiting factors that could stimulate intention and use behavior toward WFDs. Future studies are suggested to incorporate new ubiquitous factors of social influence that are perceived with the acceptance of technology by a massive population, perception of accessibility, and product value assumed by the potential users. Third, the current work utilized the factors of intention that fostered usage behavior. However, many users go through the usage behavior before developing behavioral intention. Accordingly, it would be noteworthy for future research to explore the factors of continuous intention, intention to recommend, and attitude toward WFDs in predicting the user behavior of WFDs.

## Data Availability Statement

The original contributions presented in the study are included in the article/Supplementary Material, further inquiries can be directed to the corresponding author/s.

## Ethics Statement

Ethical review and approval was not required for the study on human participants in accordance with the local legislation and institutional requirements. The patients/participants provided their written informed consent to participate in this study.

## Author Contributions

NH, GJ, MH, and AS: conceptualization, survey instrument, and writing—original draft. QY and AA: data collection, formal analysis, and writing—revision. All authors contributed to the article and approved the submitted version.

## Conflict of Interest

The authors declare that the research was conducted in the absence of any commercial or financial relationships that could be construed as a potential conflict of interest.

## Publisher's Note

All claims expressed in this article are solely those of the authors and do not necessarily represent those of their affiliated organizations, or those of the publisher, the editors and the reviewers. Any product that may be evaluated in this article, or claim that may be made by its manufacturer, is not guaranteed or endorsed by the publisher.
