# Supplementary material for: Modeling the Intention and Adoption of Wearable Fitness Devices: A Study Using SEM-PLS Analysis
Source: Front Public Health. 2022 Jul 6;10:918989. doi: 10.3389/fpubh.2022.918989 (PMC9301884; doi:10.3389/fpubh.2022.918989)
Supplement: Supplementary file 1 [file Table_1.pdf]

Supplementary Appendix 1. Survey Instrument

HCS1	I think my health depends on how well I take care of myself.	Dutta-Bergman (2009)
HCS2	I am actively engaged in the prevention of disease and illness.	
HCS3	I think taking preventive measures help to stay healthy.	
HCS4	Living a healthy life is important to me.	
HMN1	I take every action to avoid injury.	Li et al. (2019)
HMN2	I have good knowledge to prevent health issues.	
HMN3	I am confident that I can maintain my health.	
HMN4	I have good knowledge to prevent health issues.	
PCM1	Using wearable fitness devices would be compatible with my lifestyle.	Tan & Ooi (2018)
PCM2	I think that using wearable fitness devices would fit well with the way I work and live.	
PCM3	Using wearable fitness devices is compatible with all aspects of my current health care management at my personal level.	
PCM4	I think using wearable fitness devices suits my way of managing health at home.	
PCM5	I think wearable fitness device is very much compatible with my lifestyle.	
PCT1	Wearable fitness devices are cheap.	Kim, Bae & Jeon (2019)
PCT2	Prices of wearable fitness devices are reasonable.	
PCT3	Wearable fitness devices are reasonably priced.	
PCT4	I am pleased with the price of wearable fitness devices.	
PCT5	I am satisfied with the price of wearable fitness device.	
PUF1	Using wearable fitness devices makes it easier to accomplish my health condition checking.	Tan & Ooi (2018)
PUF2	Using wearable fitness device save my time and effort.	
PUF3	Wearable fitness devices are beneficial to manage health.	
PTA1	I am wearable fitness devices offers consistent results over time.	Alam et al., (2020); Walker et al. (2002)
PTA2	I think wearable fitness devices have good working standards continuously.	
PTA3	I feel confident that wearable fitness devices are offering error-free results.	
CCM1	I am using the products and services that are noticed by others.	O'Cass & McEwen (2004)
CCM2	I am using the product and services that help me to gain respect.	
CCM3	The product and services I am using show who I am.	
CCM4	I have seen other people using fitness devices.	
IWFD1	I intend to use wearable fitness devices to manage my health in the future.	Alam et al. (2020); Wang et al. (2020)
IWFD2	I plan to use wearable fitness devices frequently to manage my health in the future.	
IWFD3	I would be willing to develop a habit to use wearable fitness devices soon.	
IWFD4	I predict I will use wearable fitness devices to manage my health information.	
UWFD1	How often do you use wearable fitness devices?	Aksoy et al., (2020)

Note: HCS: Health consciousness; HMN: Health motivation; PCM: Perceived compatibility; PCT: Perceived cost; PUF: Perceived usefulness; PTA: Perceived technology accuracy; CCM: Conspicuous consumption; IWFD: Intention to Use WFD; UWFD: Usage of WFD.
Source: Author's data analysis

Supplementary Appendix 2. Discriminant Validity

	HCS	HMN	PCM	PCT	PUF	PTA	CCM	IWD	UWFD
Fornell-Larcker Criterion									
HCS	0.794								
HMN	0.720	0.802							
PCM	0.589	0.675	0.804						
PCT	0.430	0.520	0.616	0.712					
PUF	0.518	0.609	0.693	0.651	0.833				
PTA	0.444	0.549	0.675	0.645	0.698	0.812			
CCM	0.430	0.511	0.605	0.621	0.666	0.697	0.808		
IWD	0.495	0.552	0.668	0.588	0.708	0.713	0.698	0.824	
UWFD	0.351	0.427	0.541	0.491	0.522	0.553	0.622	0.601	1.000
HTMT Ratio									
HCS									
HMN	0.886								
PCM	0.706	0.805							
PCT	0.548	0.657	0.756						
PUF	0.654	0.766	0.846	0.844					
PTA	0.568	0.706	0.843	0.855	0.900				
CCM	0.529	0.626	0.717	0.777	0.834	0.894			
IWD	0.600	0.666	0.784	0.730	0.875	0.899	0.839		
UWFD	0.391	0.474	0.583	0.557	0.592	0.642	0.684	0.654	
Loading and Cross-Loading									
HCS1	*0.755*	0.511	0.431	0.334	0.387	0.295	0.304	0.354	0.255
HCS2	*0.777*	0.591	0.476	0.339	0.413	0.377	0.362	0.388	0.288
HCS3	*0.808*	0.565	0.467	0.332	0.415	0.331	0.335	0.398	0.276
HCS4	*0.834*	0.613	0.493	0.360	0.430	0.401	0.362	0.427	0.295
HMN1	0.558	*0.808*	0.547	0.412	0.478	0.444	0.424	0.443	0.355
HMN2	0.559	*0.783*	0.533	0.421	0.480	0.432	0.429	0.418	0.378
HMN3	0.607	*0.801*	0.555	0.405	0.485	0.442	0.385	0.460	0.327
HMN4	0.583	*0.814*	0.530	0.429	0.511	0.442	0.405	0.447	0.311
PCM1	0.403	0.450	*0.811*	0.560	0.505	0.443	0.421	0.436	0.295
PCM2	0.278	0.360	*0.780*	0.207	0.463	0.493	0.510	0.449	0.427
PCM3	0.278	0.346	*0.814*	0.271	0.414	0.406	0.363	0.379	0.285
PCM4	0.283	0.377	*0.791*	0.393	0.489	0.522	0.519	0.440	0.426
PCM5	0.280	0.304	*0.823*	0.167	0.437	0.417	0.374	0.375	0.295
PCT1	0.489	0.545	0.311	*0.712*	0.536	0.540	0.450	0.531	0.430
PCT2	0.485	0.555	0.280	*0.720*	0.573	0.533	0.498	0.514	0.429
PCT3	0.466	0.530	0.148	*0.721*	0.545	0.563	0.497	0.538	0.453
PCT4	0.471	0.562	0.291	*0.739*	0.568	0.535	0.491	0.555	0.409
PCT5	0.457	0.523	0.303	*0.716*	0.563	0.544	0.495	0.548	0.456
PUF1	0.414	0.504	0.576	0.570	*0.849*	0.583	0.559	0.581	0.454
PUF2	0.410	0.501	0.573	0.523	*0.785*	0.573	0.548	0.573	0.405
PUF3	0.469	0.517	0.581	0.535	*0.862*	0.587	0.557	0.613	0.444
PTA1	0.392	0.451	0.560	0.503	0.598	*0.789*	0.526	0.591	0.457
PTA2	0.387	0.445	0.556	0.523	0.568	*0.839*	0.584	0.610	0.449
PTA3	0.295	0.440	0.526	0.546	0.530	*0.807*	0.591	0.528	0.440
CCM1	0.336	0.434	0.515	0.530	0.546	0.601	*0.833*	0.558	0.541
CCM2	0.334	0.373	0.473	0.486	0.522	0.531	*0.788*	0.567	0.511
CCM3	0.337	0.411	0.453	0.499	0.511	0.559	*0.834*	0.539	0.473
CCM4	0.384	0.433	0.509	0.487	0.573	0.560	*0.773*	0.593	0.477
IWFD1	0.441	0.449	0.550	0.483	0.589	0.593	0.570	*0.840*	0.460
IWFD2	0.378	0.464	0.554	0.503	0.603	0.588	0.568	*0.839*	0.504
IWFD3	0.398	0.445	0.543	0.476	0.564	0.593	0.592	*0.783*	0.523
IWFD4	0.413	0.460	0.554	0.472	0.576	0.573	0.570	*0.831*	0.489
UWFD1	0.351	0.427	0.541	0.491	0.522	0.553	0.622	0.601	*1.000*

Note: HCS: Health consciousness; HMN: Health motivation; PCM: Perceived compatibility; PCT: Perceived cost; PUF: Perceived usefulness; PTA: Perceived technology accuracy; CCM: Conspicuous consumption; IWFD: Intention to Use WFD; UWFD: Usage of WFD.
Source: Author's data analysis
